# Genetic diversity of transmission-blocking vaccine candidate Pvs48/45 in *Plasmodium vivax* populations in China

**DOI:** 10.1186/s13071-015-1232-4

**Published:** 2015-12-01

**Authors:** Hui Feng, Bhavna Gupta, Meilian Wang, Wenqi Zheng, Li Zheng, Xiaotong Zhu, Yimei Yang, Qiang Fang, Enjie Luo, Qi Fan, Takafumi Tsuboi, Yaming Cao, Liwang Cui

**Affiliations:** Department of Immunology, College of Basic Medical Sciences, China Medical University, Shenyang, Liaoning China; Department of Entomology, Pennsylvania State University, 501 ASI Building, University Park, PA 16802 USA; Department of Microbiology and Parasitology, College of Basic Medical Sciences, China Medical University, Shenyang, Liaoning China; Department of Parasitology, College of Basic Medical Sciences, Dali Medical College, Dali, Yunnan China; Department of Parasitology, Bengbu Medical College, Anhui, China; Dalian Institute of Biotechnology, Dalian, Liaoning China; Cell-free Science and Technology Research Center, Ehime University, Matsuyama, Ehime 790-8577 Japan

**Keywords:** *Plasmodium vivax*, Transmission blocking vaccine, *pvs48/45*, Genetic polymorphism, Positive selection

## Abstract

**Background:**

The male gamete fertilization factor P48/45 in malaria parasites is a prime transmission-blocking vaccine (TBV) candidate. Efforts to develop antimalarial vaccines are often thwarted by genetic diversity of the target antigens. Here we evaluated the genetic diversity of *Pvs48/45* gene in global *Plasmodium vivax* populations.

**Methods:**

We determined 200 *Pvs48/45* sequences collected from temperate and subtropical parasite populations in China. Population genetic and evolutionary analyses were performed to determine the levels of genetic diversity, potential signature of selection, and population differentiation.

**Results:**

Analysis of the *Pvs48/45* sequences from 200 *P. vivax* parasites collected in a temperate and a tropical region revealed a low level of genetic diversity (π = 0.0012) with 14 single nucleotide polymorphisms, of which 11 were nonsynonymous. Analysis of 344 *Pvs48/45* sequences from nine worldwide *P. vivax* populations detected a total of 38 haplotypes, of which 13 haplotypes were present only once. Multiple tests for selection confirmed a signature of positive selection on *Pvs48/45* with selection skewed to the second cysteine domain. Haplotype network analysis and Wright’s fixation index showed large geographical differentiation with the presence of continent-or region-specific mutations in this gene.

**Conclusions:**

*Pvs48/45* displays low levels of genetic diversity with the presence of region-specific mutations. Some of the mutations may be potential epitope targets based on their positions in the predicted structure, highlighting the need for future evaluation of these mutations in designing Pvs48/45-based TBV.

**Electronic supplementary material:**

The online version of this article (doi:10.1186/s13071-015-1232-4) contains supplementary material, which is available to authorized users.

## Background

*Plasmodium vivax* has the widest geographical distribution of the human malarias. With as much as 19 % of the global populations being at risk of *P. vivax* infections [1–3], *P. vivax* has been increasingly recognized as a significant threat to global health. *P. vivax* is the most prevalent malaria parasites in China and distributed in both temperate and subtropical areas. The subtropical Yunnan province has year-round transmission of *P. vivax* and *P. falciparum*, whereas the central, temperate provinces such as Anhui have seasonal transmission of only *P. vivax*. A decade ago, the central provinces have experienced malaria resurgence, characterized by local outbreaks of the temperate-zone *P. vivax* malaria parasite with long relapse intervals [4–6]. This highlights the resilience of vivax malaria to control measures and underlines this parasite as a significant challenge for malaria elimination.

Interruption of malaria transmission is considered a priority task in the course of malaria elimination [7]. However, *P. vivax* produces gametocytes earlier, allowing transmission before manifestation of the symptoms in patients. In this case, transmission-blocking vaccines (TBVs) are more suitable for interrupting parasite transmission [8]. A number of candidate targets for transmission-blocking immunity such as P25 and P28 [9, 10], P48/45 [11, 12] and P230 [13, 14] have been assessed. Pfs230 and Pfs48/45 are major gametocyte and gamete surface antigens that naturally induce acquired immunity in malaria-exposed individuals [15, 16]. They are members of 6-Cys protein family [17–19], which includes additional eight members. P48/45, P47 and P230 play an essential role in gamete fertility [12, 20]. The presence of multiple disulfide bridges in the 6-Cys domains of P48/45 has hampered evaluation of the immunogenicity of the recombinant P48/45 due to difficulties to achieve proper folding of the protein. Recently, the full-length recombinant Pfs48/45 has been successfully expressed in *Escherichia coli,* which maintains functional antigenicity and induces potent transmission-blocking antibodies in mice and non-human primates [11, 21]. Similarly, sera from animals immunized with the recombinant Pvs48/45 protein or DNA vaccine also produced significant transmission blocking activity [22, 23].

Many malaria parasite antigens display extensive genetic diversity as a result of host immune selection. Genetic polymorphisms in vaccine candidates hamper vaccine development, since they tend to elicit allele variant-specific immunity, allowing immune escape mutants. Analyses performed on several *P. vivax* TBV candidates such as Pvs230 [24], Pvs48/45 [25, 26], Pvs25 and Pvs28 [27, 28] and PvWARP [25, 29] showed limited sequence diversity. To date, the majority of the analysis was done on parasites from a limited number of locations, making large-scale and comparative studies highly relevant. In this study, we compared the genetic diversity of *pvs48/45* genes from 200 clinical samples representing two distinct parasite populations in subtropical Yunnan Province and temperate-zone Anhui Province of China.

## Methods

### Collection of *P. vivax* clinical samples

Clinical *P. vivax* samples were collected from patients with acute *P. vivax* malaria in 2004 in Yunnan and in 2008–2010 in both Yunnan and Anhui provinces. Finger-prick blood samples of microscopy confirmed *P. vivax* cases were blotted onto Whatman filter papers. Informed consent was obtained from patients or their guardians, while ethical clearance for sampling collection was approved by relevant ethical committees of collaborating institutions. Use of the samples for this study was approved by the Institutional Review Board of China Medical University.

### DNA extraction, PCR and sequencing

*Plasmodium* DNA was extracted from 210 filter papers using QIAamp DNA Mini kit (Qiagen, Hilden, Germany) according to the manufacturer’s protocol. Primers and amplification of the *pvs48/45* open reading frame (ORF) were described in Additional file 1: Table S1. PCR was performed using KOD plus DNA polymerase (Toyobo, Osaka, Japan) with strong 3′ – 5′ proofreading activity. All PCR products were purified with the QIAquick Gel Extraction Kit (Qiagen, Hilden, Germany) and sequenced in both directions using the ABI Prism® BigDye™ cycle sequencing kit (Applied Biosystems, Foster City, CA, USA). Ten sequences with double peaks on the electrophoregrams suggesting of mixed infection were removed from subsequent analysis. DNA sequences obtained were assembled using the Lasergene software (DNASTAR, Madison, WI, USA) with manual editing and aligned with the Sal I reference sequence (PVX_083235) using ClustalW. Sequence data were deposited in the GenBank (KT267361-KT267560).

### Genetic diversity

Orthologs of *Pvs48/45* from other malaria parasite species *P. cynomolgi* (*Pcs48/45*; PCYB_121700), *P. knowlesi* (*Pks48/45*; PKH_120750), *P. berghei* (*Pbs48/45*; PBANKA_135960), *P. chabaudi* (*Pchs48/45*; PCHAS_136420), *P. yoelii* (*Pys48/45*; PY17X_1365300), *P. falciparum* (*Pfs48/45*; PF3D7_1346700) and *P. reichenowi* (*Prs48/45*; PRCDC_1345700) were retrieved from PlasmoDB (www.plasmodb.org). Sequences were aligned using CLUSTALW with manual editing. All the analyses in this study were done using DnaSP v5 software [30] and MEGA6 [31] except specified otherwise. A phylogenetic tree was constructed using the Neighbor-Joining method with 1000 pseudo-replications [32]. Polymorphisms were estimated by the number of single nucleotide polymorphisms (SNPs) and the average number of pairwise nucleotide differences per site (π). Distribution of π across the full-length gene was visualized by sliding window plot using a window size of 100 and step size of 25 bp. To understand differential pattern of diversity throughout the gene, *Pvs48/45* sequences were divided into three regions: i) domain I (142–483 bp), ii) domain II (892–1254 bp), and iii) inter-domain region (484–891 bp).

### Tests of neutrality

To investigate departure from neutrality, we performed Tajima’s D analysis [33, 34]. Under neutrality, Tajima’s D is expected to be 0. Significantly positive Tajima’s D values indicate recent population bottleneck or balancing selection, whereas negative values suggest population expansion or directional selection. The difference between the non-synonymous substitutions per non-synonymous site (d_N_) and numbers of synonymous substitutions per synonymous site (d_S_) was estimated using the modified Nei and Gojobori method [35]. Statistical significance of the difference was estimated using Z-test. A sliding window approach with a window size of 100 and a step size of 25 bp was used to highlight specific regions of *Pvs48/45* that deviate from neutral expectations. Five likelihood based algorithms: SLAC [36], FEL [36], IFEL [37], and REL [36] methods implemented in Datamonkey webserver [38] were used to identify the existence of positive selection pressure at individual codons. Sites were considered under positive selection if the d_N -_ d_S_ are indicated with high statistical significance (*P* <0.1 and Bayes factor >50).

We also used McDonald-Kreitman (MK) test to examine departure from neutrality [39]. The MK test compares the ratio of non-synonymous to synonymous substitutions (d_N_/d_S_) with polymorphic difference (within species; K_S_) and fixed difference (between closely related species; Ka). *Pcs48*/*45* and *Pks48*/*45* sequences were used as outgroups in this test. Fisher’s exact test was used to assess statistical significance. The null hypothesis of MK test assumes d_N_/d_S_ = Ka/Ks under neutrality, whereas d_N_/d_S_ < Ka/Ks signifies negative selection. A sliding window approach was also used to identify specific regions of *Pvs48/45* that deviate from neutral expectations using a window size of 10 and a step size of 5 bp.

### Inter-population genetic differentiation

To understand the global distribution of diversity in *Pvs48/45* gene, 200 *Pvs48/45* sequences from China were analyzed together with 144 published sequences obtained from GenBank and PlasmoDB. The geographical origins of the 344 sequences are: China (*n* = 200, this study), Thailand (*n* = 26), Korea (*n* = 40), Columbia (*n* = 28), Mexico (*n* = 15), Peru (*n* = 19), India (*n* = 3), Indonesia (*n* = 3) [26], Vanuatu (*n* = 9) and Sal I. To estimate the proportion of genetic variance due to population subdivision, Wright’s fixation index [40] of interpopulation variance in allele frequencies (Fst) was calculated. The number of haplotypes were estimated from all the isolates and the haplotype network was constructed (excluding singleton haplotypes) by NETWORK (fluxus-engineering.com) using the median joining algorithm [41].

## Results

### Divergence of P45/48 genes among *Plasmodium* species

**Fig. 1:**
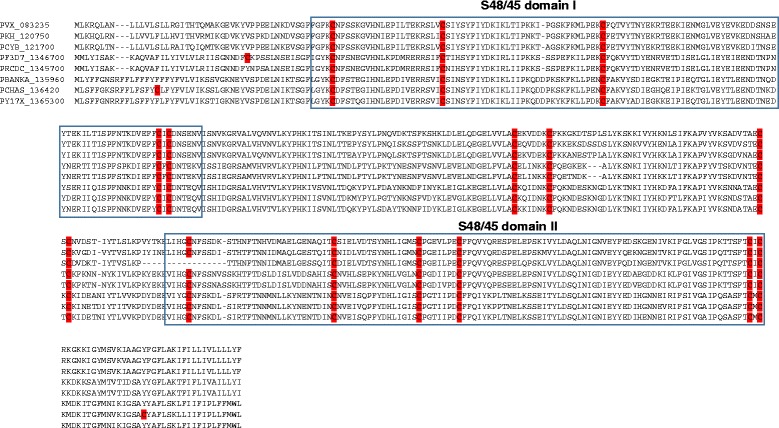
Sequence alignment of P48/45 from eight *Plasmodium* species. The s48/45 domain I and II are boxed (corresponding to the Pvx_083235 domain boundaries). Cysteine residues are highlighted in red. P48/45 amino acid sequences used are from *P. vivax* (PVX_083235), *P. cynomolgi* (PCYB_121700), *P. knowlesi* (PKH_120750), *P. berghei* (PBANKA_135960), *P. chabaudi* (PCHAS_136420), *P. yoelii* (PY17X_1365300), *P. falciparum* (PF3D7_1346700) and *P. reichenowi* (PRCDC_1345700)

**Fig. 2:**
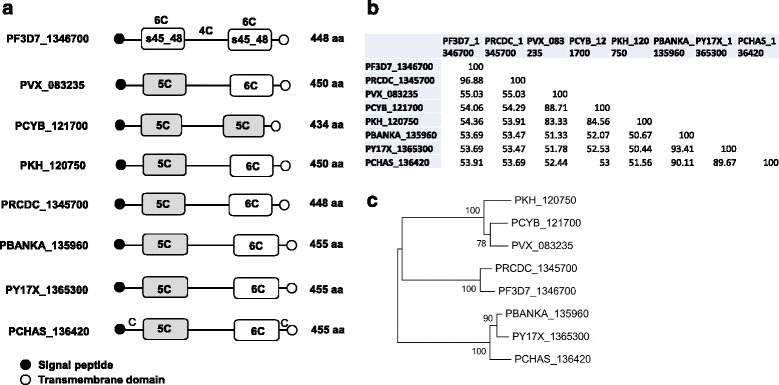
Divergence of *P48/45* gene sequence among *Plasmodium* species. **a** Schematic domain organization of P48/45 in each species. The numbers of cysteine residues in each domain are indicated. **b** Percentage of sequence similarity between amino acid sequences of eight *Plasmodium* species. **c** Neighbor-Joining tree of P48/45 amino acid sequences from eight *Plasmodium* species. Bootstrap values generated from 1000 replicates are shown

P48/45 is involved in male gamete fertility and is evolutionarily conserved in *Plasmodium* species. The full-length ORF of *Pvs48/45* is 1353 bp encoding 450 amino acids (aa), and contains two copies of the s48-45 six-Cys domain located at positions 48–162 aa and 298–418 aa, respectively. The s45-48 domain comprises of ~120 amino acids with six positionally conserved cysteine residues [42]. To understand the evolutionary history of P48/45 among *Plasmodium* species, orthologs from *P. vivax* (Sal I strain), *P. cynomolgi*, *P. knowlesi*, *P. berghei*, *P. chabaudi*, *P. yoelii*, *P. falciparum* and *P. reichenowi* were used for comparison. Alignment of these P48/45 sequences revealed 15 conserved cysteine residues (Fig. 1) with the exception of *P. cynomolgi* P48/45, which lost one residue due to a 17 bp deletion (positions 297 to 313). Of the total 15 Cys residues, five are present in s48/45 domain I, six in domain II and four in the inter-domain region (Figs. 1, 2a). Among these eight *Plasmodium* species, *P. falciparum* is the only species that contains six cysteine residues in both s48/45 domain I and domain II (Figs. 1, 2a), indicating that cysteine residue found at position 35 in domain I might have generated in the *P. falciparum* lineage after divergence from its other sister species. Moreover, *P. chabaudi* contained two more cysteine residues that are present in the region outside the s48-45 domains (Figs. 1, 2a).

The lengths of P48/45 protein sequences of the eight *Plasmodium* species varied from 455 (three rodent parasite species) to 434 residues in *P. cynomolgi* (Fig. 2a). The similarity between sequences was highest between *P. falciparum* and *P. reichenowi* (96.88 %), and lowest between *P. knowlesi* and *P. yoelii* (50.44 %) (Fig. 2b). A phylogenetic tree generated from amino acid sequences revealed three monophylectic branches, which conforms to earlier report of the phylogeny of the *Plasmodium* group based on other genetic markers [43]. *P. vivax, P. cynomolgi* and *P. knowlesi* formed a cluster, while *P. falciparum* was clustered with *P. reichenowi* (Fig. 2c). The third branch included the three rodent species.

### Genetic diversity of Pvs48/45 from the Chinese isolates

**Fig. 3:**
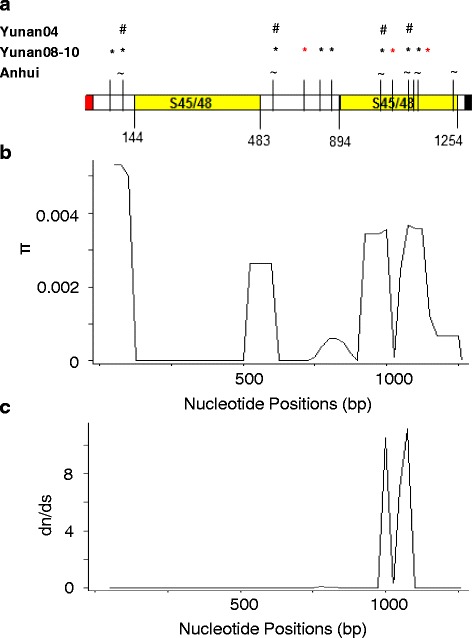
Patterns of nucleotide diversity and natural selection on *Pvs48/45*. **a** Schematic diagram of *Pvs45/48* gene showing positions of 12 SNPs (black bars) identified in the Chinese isolates. Symbols above bars represent SNPs observed in each population. The singletons have been highlighted in red. **b** Sliding window analysis of average pairwise nucleotide diversity (π). **c** dn/ds calculated using 200 *P. vivax* isolates. A window size of 100 bp and a step size of 25 bp were used for the window plot analysis

We obtained near full-length *Pvs48*/45 sequences (31–1332 bp) from 200 *P. vivax* Chinese isolates, including 61 samples collected from the temperate Anhui province in 2008–2010 and 139 samples collected from the subtropical Yunnan province. Compared to the Sal I reference sequence, there are 14 SNPs, 11 of which were non-synonymous that resulted in amino acid changes (K26R, E35K, Y196H, H211N, K250N, T273S, D335Y, A376T, I380T, G381V, and K418R) (Additional file 1: Table S2). Out of 14 SNPs, only 12 were polymorphic among 200 samples from China, while two mutations (H211N and K250N) were fixed. Four of these mutations were observed in all three populations, while six and one SNP were specific to Yunnan 08–10 and Anhui population, respectively (Fig. 3a). Three new mutations (Y196H, T273S and G381V) were identified among the Chinese isolates; the latter two were singletons.

Overall nucleotide diversity (π) in the 200 samples was 0.0012, which was similar to that observed in Yunnan 08–10, while slightly higher than those in the other two populations (Table 1). To understand the pattern of nucleotide diversity across the gene, *Pvs48/45* was divided into three blocks: two s48-45 domains separated by an inter domain. Sliding window analysis showed that these three domains were considerably different in nucleotide diversity (Fig. 3b). We identified 9 SNPs in domain II and 5 in inter-domain region, whereas s48-45 domain I was absolutely conserved in all the three populations. Nucleotide diversity was higher in domain II (π = 0.002) as compared to the inter-domain (π =0.0008) (Table 1, Fig. 3b). A similar pattern of diversity was observed in Yunnan 08–10 and Anhui samples. Among the three populations, Yunnan 08–10 samples had the highest nucleotide diversity (Table 1).

**Table 1:** Nucleotide variations and summary statistics of *Pvs48/45* in 200 *P. vivax* isolates from China

Population	*n*	S	π	Tajima’s D	d_N_-d_S_	MK (*P. cynomolgi*)	MK (*P. knowlesi*)
*Pvs48*/*45* (31–1332 bp)							
China	200	12	0.0012	−0.48702	2.028*	0.0026**	0.0126*
Yunan-2004	39	4	0.0007	−0.9270	1.676*	0.0097**	0.0247*
Yunan-2008-10	100	11	0.0012	−0.62065	1.665*	0.0065**	0.0534
Anhui-2008-10	61	6	0.0010	0.06240	2.166*	0.0009***	0.0039**
Domain I (142–483 bp)							
China	200	0	-	-	-	-	
Domain II (892–1254 bp)							
China	200	6	0.002	−0.4784	1.764*	0.0199*	NS
Yunan-2004	39	2	0.0004	−1.2936	1.380	NS	NS
Yunan-2008-10	100	5	0.0025	−0.3126	1.565	NS	NS
Anhui-2008-10	61	4	0.0021	−0.2088	1.996*	0.0131*	0.0268*
Inter-domain region (484–891 bp)							
China	200	4	0.0008	−0.9357	0.575	NS	NS
Yunan-2004	39	1	0.0011	1.3354	0.996	NS	NS
Yunan-2008-10	100	4	0.0004	−0.8926	−0.888	NS	NS
Anhui-2008-10	61	1	0.0002	−1.5282	0.992	NS	NS

*n* no. of isolates, *S* no. of SNPs, *TD*, *MK* mcdonald-kreitman test, * *P* < 0.05, **, *P* < 0.01, ***, *P* < 0.001, *NS* non-significant

Based on the amino acid sequences, a total of 15 haplotypes were observed in the 200 parasite isolates (Additional file 1: Table S3). Significant differences existed in the number of haplotypes and prevalence of individual haplotypes between the three study populations. Yunnan 08–10 samples had the highest haplotype diversity with 12 haplotypes. Three haplotypes (hap1-3) were shared among the three parasite populations. Hap2 was the most prevalent haplotype in the Yunnan 04 samples with 64.1 % prevalence, but it was rare in the Yunnan 08–10 and Anhui samples. In comparison, hap3 was much more prevalent in the Yunnan 08–10 and Anhui samples. The Yunnan 08–10 samples had seven unique haplotypes not present in the other two populations, of which hap7 reached 16.0 % prevalence.

### Departure from neutrality

Multiple tests were performed on the near full-length as well as individual blocks of *Pvs48/45* to determine whether this gene has been under natural selection. Tajima’s D values were not significant for any domains (Table 1). However, there were still differences among sites and between different domains of Pvs48/45. For example, in most cases, the Tajima’s D values were negative, suggesting the presence of rare alleles at low frequencies in these populations. Yet, in some cases, these alleles had reached higher frequencies, giving rise to a positive D value (e.g., interdomain in Yunnan 2004 samples). For the full-length sequence, d_N_ was significantly higher than d_S_ in all the populations. Likewise, d_N_/d_S_ was significantly greater than 1 in domain II of the 200 samples and the samples from Anhui. Positive selection on domain II was also demonstrated by the sliding window plot of d_N_/d_S_ values (Fig. 3c), which clearly showed d_N_/d_S_ values of >1 in s48/45 domain II, indicating positive selection on this block. This was further supported by the positively selected sites identified by the codon-based tests (Additional file 1: Table S3). Three sites were identified under positive selection by different tests, of which two are present in domain II (Additional file 1: Table S4).

**Fig. 4:**
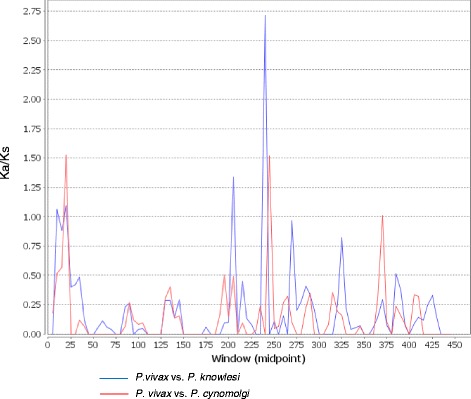
Within and between species divergence of P48/45. Sliding window analysis of Ka/Ks (non-synonymous divergence/synonymous divergence) calculated between *P. vivax* (PVX_083235) versus *P. knowlesi* (PKH_120750) and *P. cynomolgi* (PCYB_121700). A window size of 10 bp and a step size of 5 bp were used

MK test was used for comparing intraspecific polymorphism (d_N_/d_S_) and interspecific divergence (Ka/K_S_) using sequences from two phylogenetically related species *P. cynomolgi* and *P. knowlesi*. Significant values of d_N_/d_S_ > Ka/K_S_ were observed in the full-length gene, revealing excessive accumulation of synonymous substitutions between species (data not shown), which could be interpreted as negative selection for maintaining protein structure by eliminating all deleterious mutations. However, when MK test was performed on domain II and the inter-domain, this excess was significant for domain II only in the Anhui samples (Table 1). A sliding window for Ka/Ks obtained by comparing the *P. vivax* sequences to sequences of *P. cynomolgi* and *P. knowlesi* identified Ka/Ks value > 1 in signal sequence and inter-domain region (Fig. 4), thereby indicating the presence of positive selection in these two blocks.

### Geographic differentiation of worldwide *P. vivax* populations

**Fig. 5:**
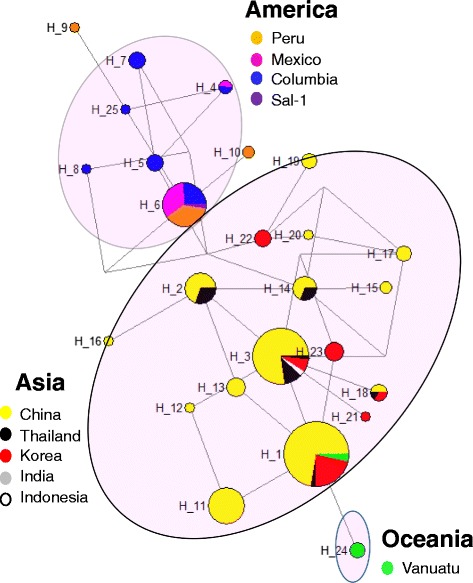
Network of the *Pvs48/45* haplotypes from global *P. vivax* populations. The size of the pies reflects the frequency of a particular haplotype. The lengths of the lines connecting the pies, measured from their centers, are in proportion to the number of base pair substitutions separating the haplotypes. Color represents different countries. Haplotypes observed in different continents are encircled

To evaluate *Pvs48/45* diversity in worldwide *P. vivax* populations, the 200 Chinese *Pvs48/45* sequences were analyzed together with 144 publically available *Pvs48*/45 sequences from nine worldwide *P. vivax* populations, including sequences from five Asian (Thailand, China, Korea, India and Indonesia), four American (Peru, Mexico, Columbia and Sal I), and one Oceania (Vanuatu) countries. Genetic differentiation among parasite populations was examined using *F*st, the Wright’s fixation index of inter-population variance in allele frequencies. The two parasite populations collected in 2008–2010 from Yunnan and Anhui provinces had low genetic differentiation (*F*st = 0.098), suggesting of extensive genetic exchanges between these populations. However, significant population differentiation was observed between the Yunnan 04 parasites and the two populations collected in 2008–2010 (*F*st = 0.290 and 0.366, respectively). Overall the *F*st estimate of the worldwide populations was 0.665, indicating that about 67 % of the variation was apportioned between parasite populations (Table 2). Pairwise comparisons between populations revealed a wide range of *F*st values (0.34 – 0.90) between populations from different continents compared to that observed within continents (0.01 – 0.43) (Table 2). Genetic differentiation between populations relative to each continent was also evident from the haplotype network constructed from the worldwide haplotypes (Fig. 5). A total of 38 haplotypes were identified within the 344 sequences, of which 13 singleton haplotypes (observed only once) were excluded from the analysis. The clustered distribution of the haplotypes relevant to the continent of origin is apparent from the haplotype network (Fig. 5).

**Table 2:** Pairwise F_ST_ estimates for nine worldwide *Plasmodium vivax* populations using *Pvs48* gene sequences

	Thailand	Columbia	Mexico	Peru	China	Korea	Vanuatu	Indonesia	India
Thailand									
Columbi	0.57								
Mexico	0.75	0.39							
Peru	0.75	0.43	0.03						
China	0.11	0.64	0.80	0.79					
Korea	0.18	0.64	0.79	0.78	0.04				
Vanuatu	0.49	0.78	0.90	0.89	0.34	0.32			
Indonesia	0.09	0.69	0.83	0.83	0.01	0.04	0.36		
India	0.27	0.75	0.89	0.88	0.37	0.38	0.68	0.25

## Discussion

Host immunity plays an important role in shaping the genetic repertoire of malaria parasite antigens. Understanding genetic diversity of these antigens is essential for designing antimalarial vaccines [44, 45]. In the present study, we analyzed 200 *Pvs48/45* sequences of clinical *P. vivax* samples from two geographical regions of China and compared them with *Pvs48/45* sequences from other endemic regions. The phylogenetic tree based on P48/45 sequences agreed well with those constructed with the mitochondrial genomes and 18 s rRNA genes [43, 46]. Despite the levels of sequence similarity between species varied from 50.44 % to 96.88 %, the positions of cysteine residues in the gene were highly conserved, supporting a generalizing feature of the 6-Cys gene family. Within *P. vivax*, 11 amino acid changes were observed between the Chinese samples and the Sal I reference strain, while two of them were fixed among the 200 isolates from China (Additional file 1: Table S2). In addition, seven of the 11 amino acid changes were parsimony informative (observed in more than one sequence). Eight of these mutations are previously known from other Asian countries [25, 26], while one novel mutation (Y196H) was observed in all the three populations from China. Mutations H211N, K250N and K418R have been previously identified as important targets for vaccine design based on their structural positions [26]. In our populations, mutations H211N and K250N are fixed while K418R is present in 97 % of the total isolates (193 out of 200).

Like other sexual stage antigens, *Pvs48/45* exhibited low levels of genetic diversity. The measure of nucleotide diversity (0.0012) is in a similar range with other sexual stage antigens such as *Pvs25* (0.0013) in China [28] and worldwide *Pvs230* (0.00118) [24]. Pvs48/45 was even more conserved in the Korean populations where nucleotide diversity varied from 0.00147 [26] to 0.00053 [25]. The level of genetic diversity was at par between the two parasite populations in China, whereas parasites from Yunnan province appeared to have increased genetic diversity over the years (Table 1). Interestingly, however, seven out of 11 amino acid changes commonly observed in the samples collected at different time intervals were either fixed or highly frequent (frequency varying from 36-100 %) except Y196H mutation, which was less frequent in the later populations collected in 2008–10. Four mutations that were observed only in 2008–10 were rare with frequency varying from 1 % to 7 %. These mutations might be the result of difference in the number of samples collected at the two time points and/or recently increased parasite introduction from neighboring malaria endemic countries due to heightened cross-country human migration. This similar pattern of diversity in space and time further suggests that the low-level genetic diversity in *Pvs48/45* seems to be imposed by natural selection acting to maintain the functional/structural characteristics of this protein.

Immunoepidemiological studies of sexual stage, pre-fertilization, antigens such as P230 and P48/45 showed that antibody responses to these proteins are present in endemic human populations, and are associated with transmission blocking activities [16, 47–49]. As such, it is expected that these antigens are under host immune selection, which may lead to significant genetic polymorphisms in antigens. Previous studies suggested positive selection on sexual stage antigens such as the male and female gamete fertilization factors *Pfs47* and *Pfs48/45* [50]. Similarly, our data demonstrated positive selection on *Pvs48/45*. Moreover, distribution of the polymorphic sites is not even across the gene, but rather concentrated in domain II of this gene and domain I was monomorphic. This might be due to the differential selection pressure acting on two domains because of their specific functions or differential exposure to host immunity [51]. On the other hand, Ka/KS rate showed values greater than 1 in regions outside of the two s48-45 domains. This inter-species pattern of selection might be the consequence of long-term evolution of each species within their respective hosts. This is in contrast to another 6-Cys, gamete-surface antigen pvs230, which was found to be under purifying selection [24]. Such divergent selections on two male gamete surface proteins might be due to differences in their functional constraints, as P48/45 is involved in binding to female gametes [12] and P230 mediates binding of red blood cells [52]. It is also noteworthy that the MK analysis showed significant accumulation of inter-species synonymous substitutions, suggesting that *Pvs48/45* might have diverged from *P. knowlesi* and *P. cynomolgi* due to negative selection acting on deleterious mutations. Similar patterns of evolution have been reported in other members of 6-Cys family [24, 53].

In *P. falciparum*, merozoite antigens have high levels of diversity globally but less geographical isolation possibly due to host immune selection [45]. Data obtained in *P. vivax* such as PvTRAP, PvDBP and PvAMA-1 also supported such a conclusion [54]. In contrast, non-merozoite antigens such as sexual stage antigen Pfs48/45 showed significant geographical differentiation, possibly as a result of gene flow barriers or/and divergent selection on the amino acid sequences of these proteins in different populations [55]. Similarly, analysis of worldwide *Pvs48/45* sequences revealed evident genetic structure between geographical parasite populations as shown by relatively high fixation indices. Inter-continent *F*_ST_ values are much higher than those of parasites within a continent. In China, despite the geographical separation of Yunnan and Anhui provinces, the prevalence of different mutations and haplotypes were similar between the two parasite populations collected in recent years (2008–2010) from these two localities, with differences being present only in rare alleles such as the singleton mutations. However, compared with the earlier parasite population collected in Yunnan in 2004, there were major allele changes, reflected in the emergence of the I380T mutations in the later populations and significantly reduced prevalence of the Y196H mutations. Taken together, although worldwide *Pvs48/45* genes displayed a high-level sequence conservation, continent-or region-specific mutations exist in different population, especially in the second s48-45 domain, which was apparently under positive selection. Therefore, precautions need to be taken when designing TBV against Pvs48/45.

## Conclusions

Pvs48/45 displays low levels of genetic diversity with the presence of region-specific mutations. Some of the mutations may be potential epitope targets based on their positions in the predicted structure, highlighting the need for future evaluation of these mutations in designing Pvs48/45-based TBV.

## Additional file

Additional file 1: Table S1. Primers used for PCR and sequencing of *Pvs48/45*. **Table S2.** Nonsynonymous mutations and their frequencies [n (%)] in different parasite populations collected in China. **Table S3.** Pvs48/45 haplotypes and their frequencies in Anhui and Yunnan provinces. **Table S4.** Selection pressure analysis of the 200 *Pvs48/45* sequences (434 codons) using the SLAC, FEL, IFEL and REL methods employed in the Datamonkey server. (DOC 73 kb)
