# Supplementary material for: Genetic diversity of transmission-blocking vaccine candidate Pvs48/45 in Plasmodium vivax populations in China
Source: Parasit Vectors. 2015 Dec 1;8:615. doi: 10.1186/s13071-015-1232-4 (PMC4665908; doi:10.1186/s13071-015-1232-4)
Supplement: Additional file 1: Table S1. — Primers used for PCR and sequencing of Pvs48/45. Table S2. Nonsynonymous mutations and their frequencies [n (%)] in different parasite populations collected in China. Table S3. Pvs48/45 haplotypes and their frequencies in Anhui and Yunnan provinces. Table S4. Selection pressure analysis of the 200 Pvs48/45 sequences (434 codons) using the SLAC, FEL, IFEL and REL methods employed in the Datamonkey server. (DOC 73 kb) [file 13071_2015_1232_MOESM1_ESM.doc]

**Supplementary Table 1**

*Primers used for PCR and sequencing of Pvs48/45*

| **Primers** | **Sequences (5’ 🡪3’)** | **Purpose** |
| --- | --- | --- |
| pvs48F | GTTTAAACTTAAGCTTATGTTGAAGCGCCAGCTCGC | PCR |
| pvs48R | GCCCTCTAGACTCGAGTCAGAAGTACAACAGGAGGAG | PCR |
| *pvs48*NestF | GCTCGCCAACCTGCTGCTCGTGCTG | Nested PCR |
| *pvs48*NestR | CCTCATTGTGCTCCTCCTGTTGTAC | Nested PCR |
| *pvs48* seqF | GAACCATATTCTTACCTACC | Sequencing |
| *pvs48* seqR | AGGAGCATTCTGCGGTCACG | Sequencing |

The PCR reaction contained 2 μl of 10×KOD-Plus-Neo buffer, 2 μl of 2 mM dNTPs, 0.8 μl of 25 mM MgSO_4_, 0.5 μl of 10 μM of each primer, 0.5 units of KOD-Plus-Neo DNA polymerase (Toyobo, Osaka, Japan), and 1.0 μl genomic DNA template in a final volume of 20 μl. Amplification conditions were as follows: initial denaturation at 94°C for 2 min, 35 cycles of 94°C for 15 sec, 56°C for 15 sec, and 68°C for 90 sec, and a final extension at 68°C for 5 min.

**Supplementary Table 2**

Nonsynonymous mutations and their frequencies [n (%)] in different parasite populations collected in China.

| **Mutations*** | **Yunnan 04** | **Yunnan 08-10** | **Anhui 08-10** |
| --- | --- | --- | --- |
| K26R | - | 3 (3%) | - |
| E35K | 32 (82.1%) | 36 (36%) | 38 (62.3%) |
| Y196H | 26 (66.7%) | 3 (3%) | 2 (3.3%) |
| H211N | 39 (100%) | 100 (100%) | 61 (100%) |
| K250N | 39 (100%) | 100 (100%) | 61 (100%) |
| T273S | - | 1 (1%) | - |
| D335Y | 37 (94.9%) | 63 (63%) | 56 (91.8%) |
| A376T | 38 (97.4%) | 81 (81%) | 54 (88.5%) |
| I380T | - | 5 (5%) | 7 (11.5%) |
| G381V | - | 1 (1%) | - |
| K418R | 39 (100%) | 100 (100%) | 54 (88.5%) |
| Total samples | 39 | 100 | 61 |

* Mutations were compared with the Sal I reference sequence.

**Supplementary Table 3**

Pvs48/45 haplotypes and their frequencies in Anhui and Yunnan provinces.

|  | Sequence at amino acid 26/35/196/273/335/376/380/381/418 | Yunnan 04 (n=39) | Yunnan 08-10 (n=100) | Anhui 08-10 (n=61) |
| --- | --- | --- | --- | --- |
| Hap1 | KEYNNTYTIGR | 5 (12.8%) | 26 (26.0%) | 20 (32.8%) |
| Hap2 | KKHNNTYTIGR | 25 (64.1%) | 2 (2.0%) | 1 (1.6%) |
| Hap3 | KKYNNTYTIGR | 6 (15.4%) | 25 (25.0%) | 32 (52.5%) |
| Hap4 | KEHNNTYTIGR | 1 (2.6%) | 0 | 1 (1.6%) |
| Hap5 | KKYNNTDTIGR | 1 (2.6%) | 7 (7.0%) | 0 |
| Hap6 | KEYNNTDAIGR | 1 (2.6%) | 12 (12.0%) | 0 |
| Hap7 | KEYNNTDTIGR | 0 | 16 (16.0%) | 0 |
| Hap8 | KEYNNTYAIGR | 0 | 1 (1.0%) | 0 |
| Hap9 | KEYNNTYATGR | 0 | 5 (5.0%) | 0 |
| Hap10 | KKYNNTDAIGR | 0 | 1 (1.0%) | 0 |
| Hap11 | REYNNTYTIGR | 0 | 3 (3.0%) | 0 |
| Hap12 | KEYNNSDTIGR | 0 | 1 (1.0%) | 0 |
| Hap13 | KKHNNTYTIVR | 0 | 1 (1.0%) | 0 |
| Hap14 | KKYNNTDATGK | 0 | 0 | 5 (8.2%) |
| Hap15 | KEYNNTYATGK | 0 | 0 | 2 (3.3%) |
| Haplotype diversity |  | 0.561 | 0.842 | 0.619 |

**Supplementary Table 4**

**Selection pressure analysis of the 200 *Pvs48/45* sequences (434 codons) using the SLAC, FEL, IFEL and REL methods employed in the Datamonkey server.**

| Positively selected Codons | SLAC  (P <0.1) | FEL  (P <0.1) | IFEL  (P <0.1) | REL  (Bayes factor>50) | Genetic region |
| --- | --- | --- | --- | --- | --- |
| E35K | - | 0.081 | - | 154.22 | N-terminal |
| Y335D | - | - | 0.092 | 151.775 | s48/45 domain II |
| T376A | - | - | 0.079 | 154.178 | s48/45 domain II |
